# Supplementary material for: Density-Based Separation of Microbial Functional Groups in Activated Sludge
Source: Int J Environ Res Public Health. 2020 Jan 6;17(1):376. doi: 10.3390/ijerph17010376 (PMC6981482; doi:10.3390/ijerph17010376)
Supplement: Supplementary file 1 [file ijerph-17-00376-s001.pdf]

Supplementary Information

Density-based Separation of Microbial Functional Groups in Activated Sludge

Lin Li, Yaqi You and Krishna Pagilla *

Department of Civil and Environmental Engineering, University of Nevada, Reno, NV 89557, USA; lli234@nevada.unr.edu (L.L.); you.yaqi@gmail.com (Y.Y.)
* Correspondence: pagilla@unr.edu; Tel.: +1-775-682-7918

Supplementary Materials and Methods

1. Activated Sludge Characterization and Concentration.

Activated sludge (AS) samples were characterized for total solids (TS), volatile solids (VS), total suspended solids (TSS) and volatile suspended solids (VSS), according to Standard Methods (23rd edition) [1]. Floc sizes and filament abundance were determined by microscopic imaging according to Tandoi et al. (2017) and Jarvis et al. (2005) [2,3]. Before density-based separation, samples were concentrated by serial centrifugation, each round at 3000 rpm for 10 min, in order to keep TS around 20 $\times 10^{-3}$ g/cm^3. Finally, condensed samples were vortexed for 1 min for homogenization.

2. PCR Amplification

Conventional PCR was conducted on a BioRad C1000 Touch Thermal Cycler (BioRad, Hercules, CA, USA) using primer sets and annealing temperatures listed in Table 2. Reactions were performed in 25 μL volume, containing 1× *Taq* reaction buffer, 1 U of *ExTaq* DNA polymerase (TaKaRa), 0.48 μM each primer, 0.2 mM each dNTP, and 10 to 20 ng of the template. The PCR program consisted of initial denaturation at 94 °C for 5 min, with 30-35 cycles of 94 °C for 45 sec, the annealing temperature (Table 2) for 1 min, and 72 °C for 60 sec, followed by a final extension at 72 °C for 10 min. Amplicons were verified by electrophoresis.

Both positive and negative controls were included in each PCR run. Positive controls included sequenced PCR amplicons, and negative controls included ultrapure water. Examination of inhibitory substances was performed as before [4,5]. Briefly, 10-fold serial dilutions of environmental DNA were used as template with the primer pair 27F/1492R that amplifies nearly complete 16S rRNA gene of eubacteria [6]. The dilution resulting in a bright gel band was considered as the appropriate dilution factor for mitigating inhibitors co-extracted from sludge biomass matrix.

3. PCR Product Cleanup, Cloning and Plasmid Analysis

PCR products were purified by QIAprep Spin Miniprep Kit (Qiagen, Germantown, MD, USA) according to the manufacturer's protocol and sequenced on both strands using PCR primers as detailed below. After verification of their identities by sequencing, PCR products were cloned using TOPO TA Cloning kit (Invitrogen, Carlsbad, CA, USA) on the pCR4-TOPO vector and transformed into One Shot Competent *Escherichia coli*, in accordance with the manufacturer's protocol. For each PCR product, 10 to 15 clones were randomly selected for plasmid analysis.

Plasmids were extracted from overnight cultures using QIAprep Spin Miniprep kit (Qiagene, Germantown, MD, USA) and subjected to restriction digestion with *Eco*RI (Promega, Madison, WI, USA) according to the manufacturer's protocol with minor modifications. Restriction fragments were separated by agarose gel electrophoresis for size approximations. Plasmids were also sequenced to confirm correct constructs (detailed below).

4. qPCR Accuracy

Calibration curves (4 to 6 log range in duplicate or triplicate) were generated with 10-fold serial dilutions of cloned PCR products. Correlation coefficients (R^2) > 0.99 were obtained for all calibration curves, with amplification efficiencies of 91% to 96% (Table 2). Detection limits were < 1.61×10^3 copies for eubacterial 16S rRNA genes, < 9.72×10^4 for PAO 16S rRNA genes, < 6.75×10^2 for *Nitrosomonas* spp. amoA genes, < 2.73×10^4 for *Nitrospira* spp. 16S rRNA genes, and < 3.34×10^4 for *Nitrobacter* spp. 16S rRNA genes. While these detection limits were not as low as some other reports, the assays served well for the quantification purpose in this study.

After the predetermination of appropriate dilution factors for mitigating PCR inhibition by sludge biomass matrix, all biomass DNA samples were diluted for 50- and 100-fold in 2–3 independent replicates. Then 2 uL of the diluted sample was used as template in reactions. Besides melt curve analysis, selected qPCR products were also verified by gel electrophoresis and sequencing.

For calculating the relative abundance of each microbial functional group, copy numbers of each target gene were normalized against eubacterial 16S rRNA gene copy numbers, with the following assumptions: (1) 4.1 copies of the *rrn* operon per eubacterial genome [7–10], (2) 2 copies of the *rrn* operon per *Ca.* Accumulibacter genome [8,10,11], (3) 2 copies of *amoA* gene per *Nitrosomonas* spp. genome [7,12,13], and (4) 1 copy of the *rrn* operon per *Nitrospira* spp. genome and per *Nitrobacter* spp. genome [7,14–16].

5. DNA Sequencing and Sequence Analysis

PCR products and plasmid clones purified from *E. coli* transformants were sequenced on both strands using an Applied Biosystems 3730xl DNA Analyzer at Eurofin Genomics (Louisville, KY, USA). PCR products were sequenced using the PCR primers. Plasmids were sequenced with the M13F and M13R universal primers. Sequences were manually checked, and the forward and reverse strands were aligned using MegAlign (DNASTRA) to obtain the complete reads. All sequences of the same gene were aligned using ClustalX 2.0 [17] and trimmed to the same length without primer binding sites. MEGA6 was used to generate neighbor-joining trees with the Tamura-Nei mode [18]. The reliability of the resulting trees was assessed by bootstrap analysis of 1,000 replicates.

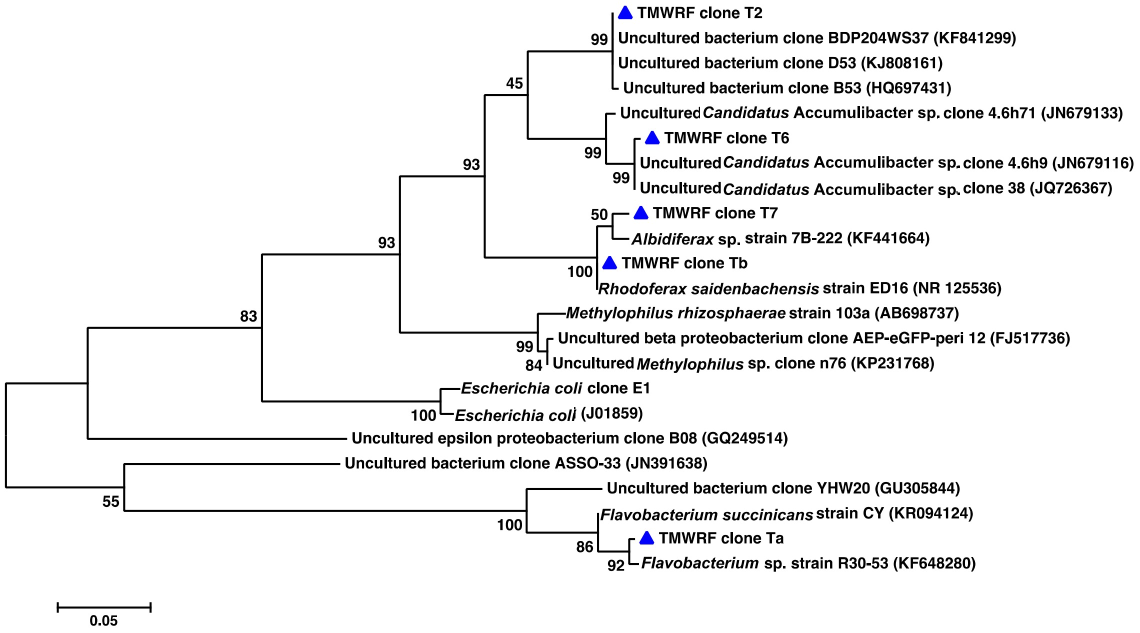

Figure S1. Maximum likelihood tree of representative bacterial taxa identified in AS from the EBPR facility TMWRF. 16S rRNA genes were amplified from environmental DNA, cloned on the pCR4-TOPO vector, and sequenced; sequences were trimmed to the same length excluding primer biding sites. The tree was constructed using the Tamura-Nei model. Bootstrap resampling (1,000 replicates) support values are shown next to the branches. The bar represents 0.05 nucleotide substitutions per site. GenBank accession numbers of published sequences are listed. Sequences identified in this study are denoted by a blue triangle.

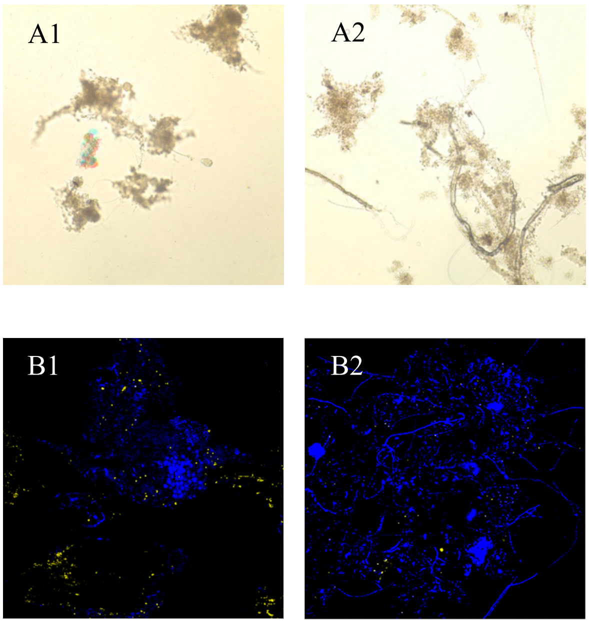

Figure S2. Representative phase contrast images (A) and CLSM images (B) of flocs in unseparated activated sludge of (1) TMWRF aeration tank and (2) STMWRF oxidation ditch. Based on measurements of 20 flocs for each WRRF, the average size of flocs in TMWRF and STMWRF was determined to be approximately 40 µm and 60 µm, respectively. TMWRF sludge showed round, compact flocs, and microbes therein tended to cluster together. STMWRF sludge had more filamentous microbes and thus a relatively loose structure.

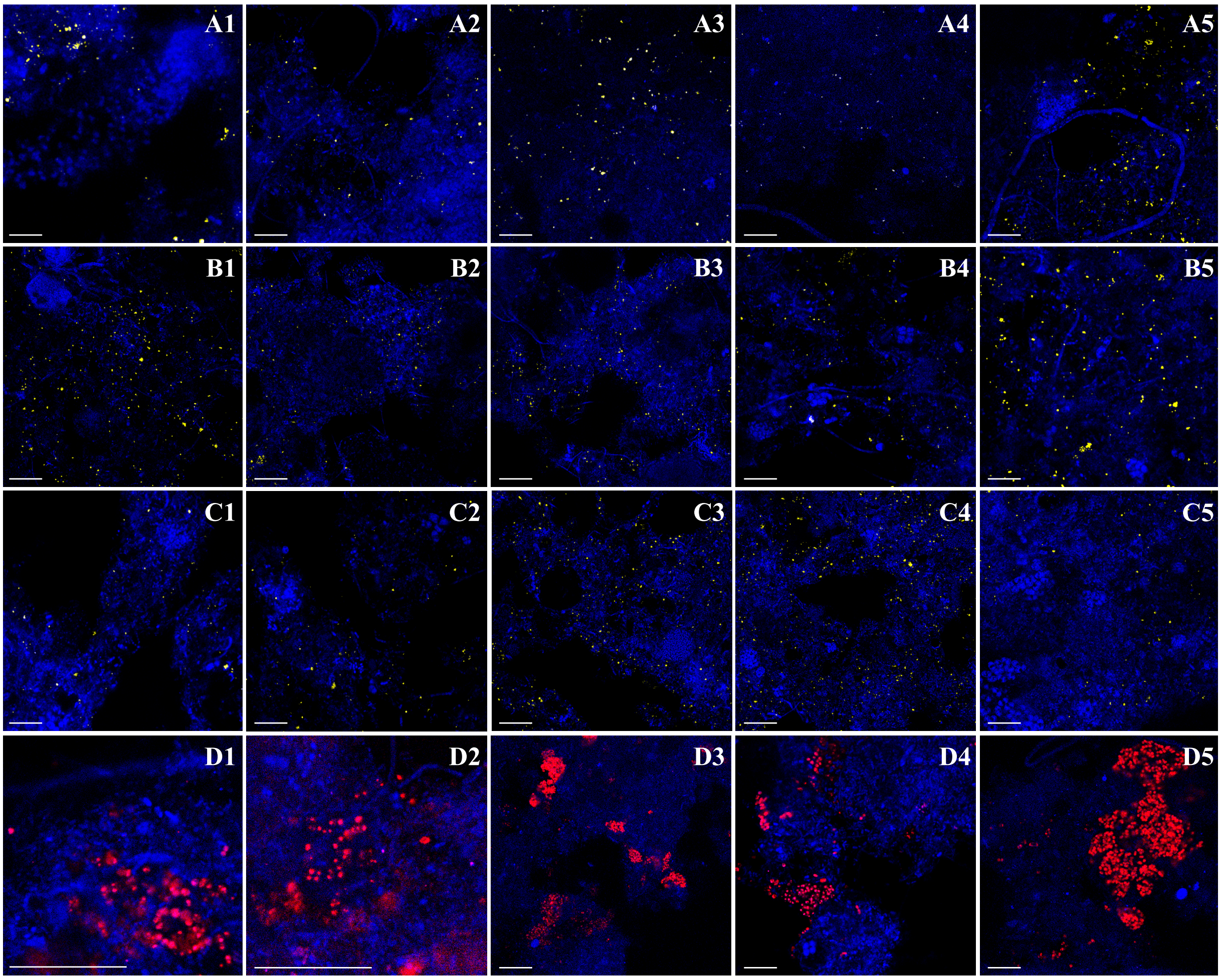

Figure S3. CLSM images of morphology and spatial organization of sludge containing (A) AOB (yellow), (B) *Nitrobacter* spp. NOB (yellow), (C) *Nitrospira* spp. NOB (yellow), and (D) PAOs (red), along with total eubacteria (blue). Shown here are (1) unseparated sludge, (2) sludge fraction lighter than 1.030 g/cm^3, (3) sludge fraction denser than 1.030 g/cm^3, (4) sludge fraction lighter than 1.036 g/cm^3, and (5) sludge fraction denser than 1.036 g/cm^3, all from TMWRF aeration tank. Scale bar indicates 20 μm.

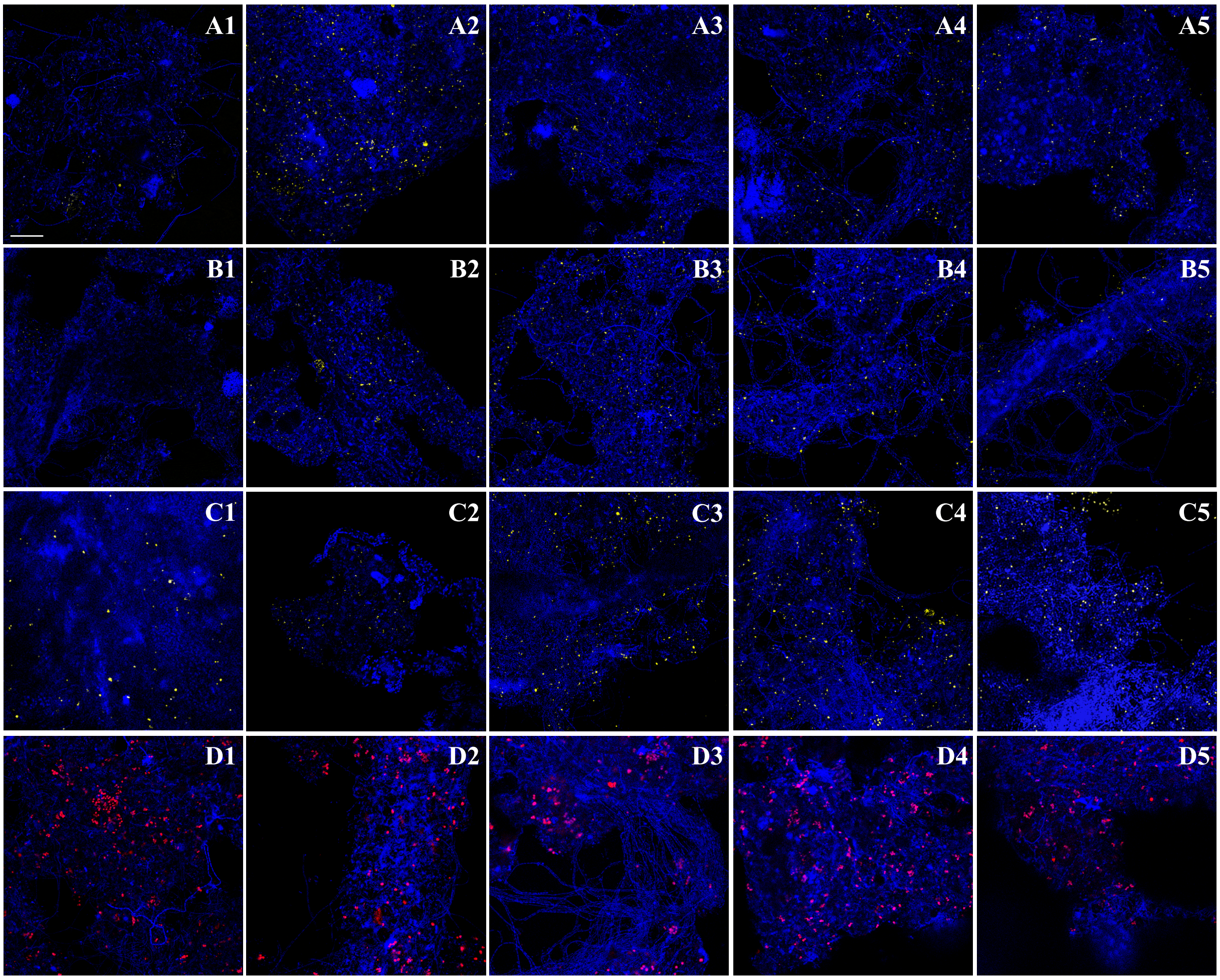

Figure S4. CLSM images of morphology and spatial organization of sludge containing (A) AOB (yellow), (B) *Nitrobacter* spp. NOB (yellow), (C) *Nitrospira* spp. NOB (yellow), and (D) PAOs (red), along with total eubacteria (blue). Shown here are (1) unseparated sludge, (2) sludge fraction lighter than 1.030 g/cm^3, (3) sludge fraction denser than 1.030 g/cm^3, (4) sludge fraction lighter than 1.036 g/cm^3, and (5) sludge fraction denser than 1.036 g/cm^3, all from STMWRF oxidation ditch. Scale bar indicates 20 μm.

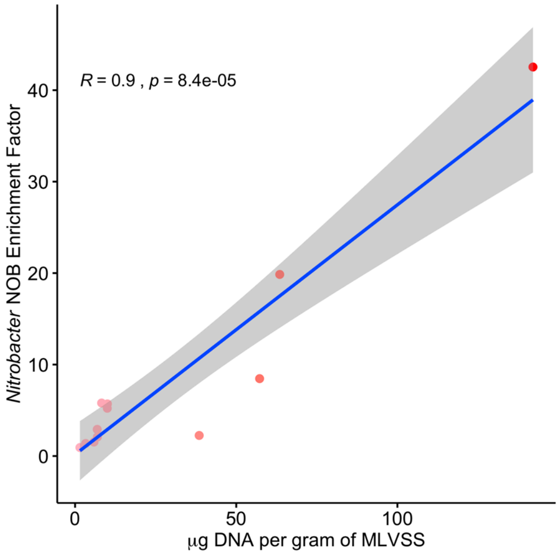

Figure S5. Enrichment of *Nitrobacte*r spp. NOB was significantly correlated with DNA amount per gram of MLVSS in AS, as shown by Spearman rank correlation test.

References

1. Rice, E.; Baird, R.; Eaton, A. *Standard Methods for the Examination of Water and Wastewater*; 23rd ed.; American Water Works Association, American Public Health Association, Water Environment Federation, 2017;
2. Jarvis, P.; Jefferson, B.; Gregory, J.; Parsons, S.A. A review of floc strength and breakage. *Water Res.* **2005**, *39*, 3121–3137.
3. Tandoi, V.; Rossetti, S.; Wanner, J. *Activated Sludge Separation Problems: Theory, Control Measures, Practical Experiences*; IWA Publishing, 2017; ISBN 978-1-78040-863-7.
4. Harms, G.; Layton, A.C.; Dionisi, H.M.; Gregory, I.R.; Garrett, V.M.; Hawkins, S.A.; Robinson, K.G.; Sayler, G.S. Real-Time PCR Quantification of Nitrifying Bacteria in a Municipal Wastewater Treatment Plant. *Environ. Sci. Technol.* **2003**, *37*, 343–351.
5. You, Y.; Hilpert, M.; Ward, M.J. Detection of a Common and Persistent tet(L)-Carrying Plasmid in Chicken-Waste-Impacted Farm Soil. *Appl Env. Microbiol* **2012**, *78*, 3203–3213.
6. Weisburg, W.G.; Barns, S.M.; Pelletier, D.A.; Lane, D.J. 16S ribosomal DNA amplification for phylogenetic study. *J. Bacteriol.* **1991**, *173*, 697–703.
7. Dionisi, H.M.; Layton, A.C.; Harms, G.; Gregory, I.R.; Robinson, K.G.; Sayler, G.S. Quantification of Nitrosomonas oligotropha-Like Ammonia-Oxidizing Bacteria and Nitrospira spp. from Full-Scale Wastewater Treatment Plants by Competitive PCR. *Appl. Environ. Microbiol.* **2002**, *68*, 245–253.
8. He, S.; Gall, D.L.; McMahon, K.D. "Candidatus Accumulibacter" Population Structure in Enhanced Biological Phosphorus Removal Sludges as Revealed by Polyphosphate Kinase Genes. *Appl. Environ. Microbiol.* **2007**, *73*, 5865–5874.
9. Větrovský, T.; Baldrian, P. The Variability of the 16S rRNA Gene in Bacterial Genomes and Its Consequences for Bacterial Community Analyses. *PLOS ONE* **2013**, *8*, e57923.
10. Mao, Y.; Graham, D.W.; Tamaki, H.; Zhang, T. Dominant and novel clades of *Candidatus* Accumulibacter phosphatis in 18 globally distributed full-scale wastewater treatment plants. *Sci. Rep.* **2015**, *5*, 11857.
11. Martín, H.G.; Ivanova, N.; Kunin, V.; Warnecke, F.; Barry, K.W.; McHardy, A.C.; Yeates, C.; He, S.; Salamov, A.A.; Szeto, E.; et al. Metagenomic analysis of two enhanced biological phosphorus removal (EBPR) sludge communities. *Nat. Biotechnol.* **2006**, *24*, 1263.
12. Kowalchuk, G.A.; Stephen, J.R. Ammonia-Oxidizing Bacteria: A Model for Molecular Microbial Ecology. *Annu. Rev. Microbiol.* **2001**, *55*, 485–529.

13. Chain, P.; Lamerdin, J.; Larimer, F.; Regala, W.; Lao, V.; Land, M.; Hauser, L.; Hooper, A.; Klotz, M.; Norton, J.; et al. Complete Genome Sequence of the Ammonia-Oxidizing Bacterium and Obligate Chemolithoautotroph Nitrosomonas europaea. *J. Bacteriol.* **2003**, *185*, 2759–2773.
14. Navarro, E.; Fernandez, M.P.; Grimont, F.; Clays-Josserand, A.; Bardin, R. Genomic Heterogeneity of the Genus Nitrobacter. *Int. J. Syst. Evol. Microbiol.* **1992**, *42*, 554–560.
15. Starkenburg, S.R.; Chain, P.S.G.; Sayavedra-Soto, L.A.; Hauser, L.; Land, M.L.; Larimer, F.W.; Malfatti, S.A.; Klotz, M.G.; Bottomley, P.J.; Arp, D.J.; et al. Genome Sequence of the Chemolithoautotrophic Nitrite-Oxidizing Bacterium Nitrobacter winogradskyi Nb-255. *Appl Env. Microbiol* **2006**, *72*, 2050–2063.
16. Daims, H.; Lebedeva, E.V.; Pjevac, P.; Han, P.; Herbold, C.; Albertsen, M.; Jehmlich, N.; Palatinszky, M.; Vierheilig, J.; Bulaev, A.; et al. Complete nitrification by Nitrospira bacteria. *Nature* **2015**, *528*, 504–509.
17. Larkin, M.A.; Blackshields, G.; Brown, N.P.; Chenna, R.; McGettigan, P.A.; McWilliam, H.; Valentin, F.; Wallace, I.M.; Wilm, A.; Lopez, R.; et al. Clustal W and Clustal X version 2.0. *Bioinformatics* **2007**, *23*, 2947–2948.
18. Tamura, K.; Stecher, G.; Peterson, D.; Filipski, A.; Kumar, S. MEGA6: Molecular Evolutionary Genetics Analysis Version 6.0. *Mol. Biol. Evol.* **2013**, *30*, 2725–2729.
